# LKB1-AMPK-mTOR信号传导通路在肿瘤中的研究进展

**DOI:** 10.3779/j.issn.1009-3419.2011.08.09

**Published:** 2011-08-20

**Authors:** 霞 张

**Affiliations:** 1 300052 天津，天津医科大学总医院呼吸科 Department of Respiratory Medicine, Tianjin Medical University General Hospital, Tianjin 300052, China; 2 天津医科大学总医院，天津市肺癌研究所，天津市肺癌转移与肿瘤微环境实验室 Tianjin Key Laboratory of Lung Cancer Metastasis and Tumor Microenvironment, Tianjin Lung Cancer Institute, Tianjin Medical University General Hospital, Tianjin 300052, China

肿瘤可以认为是一种基因疾病，其中癌基因的激活和抑癌基因的失活是肿瘤发生、发展过程中最为关键的环节之一^[1]^。近年来，一个新的抑癌基因*LKB1*，又名*STK11*(serine threonine protein kinase 11)，在肿瘤中的作用引起了越来越多的关注。哺乳动物雷帕霉素靶蛋白(mammalian target of rapamycin, mTOR)信号通路是目前肿瘤信号传导通路研究的热点之一。研究^[2]^显示LKB1通过磷酸化磷酸腺苷激活的蛋白激酶(AMP-activated protein kinase, AMPK)，从而激活AMPK，实现对mTOR活性的负向调控。本文就LKB1-AMPK-mTOR信号通路及其在肿瘤中的研究进展进行简要的综述。

## 1 LKB1的结构与功能

*LKB1*基因位于人的第19号染色体短臂13.3区，包含9个编码的外显子和1个非编码外显子，其编码的LKB1蛋白由433个氨基酸组成，属于丝氨酸/苏氨酸蛋白激酶，第44-309位氨基酸为激酶催化区，N端第38-43位氨基酸残基是核定位信号序列(nuclear localization signal, NLS)，该序列的缺失将导致LKB1遍布整个细胞，但不影响其抑制细胞生长的功能。LKB1蛋白主要定位于细胞核，细胞浆中只有少量表达，但其功能主要与细胞浆内的部分有关^[3]^。LKB1与STRAD(STE20 related adaptor protein)和MO25(mouse protein 25)蛋白形成复合体，可极大地提高其激酶活性。STRAD蛋白缺少催化蛋白质磷酸化所必须的关键残基(即Vib和VII两个模体)，是一个假激酶，当STRAD与LKB1形成复合体后，可促进后者从细胞核内移位到细胞浆内^[4]^；MO25蛋白与STRAD羧基端结合，增加了LKB1-STRAD复合物在细胞浆中的空间定位和构象，使LKB1的活性提高了近10倍^[5]^。Rowan等^[6]^研究表明，人体中几乎所有的组织均有LKB1 mRNA的表达，其中胎儿组织中的表达高于成人，成人以上皮、睾丸生精小管和肝脏表达最强。

*LKB1*基因的胚系突变(germline mutation)是黑斑息肉综合征(Peutz-Jeghers syndrome, PJS)的主要致病原因^[7]^。PJS是一种以胃肠道多发性错构瘤息肉样改变和黏膜色素沉着为特征的常染色体显性遗传疾病，该类患者的肿瘤发生率是普通人群的10倍-18倍^[8]^，以消化道肿瘤最常见，还可伴发其它部位，如乳腺、子宫、卵巢、睾丸和胰腺等的肿瘤^[9]^。66%-94%的PJS患者可以检测到*LKB1*基因突变，突变类型以点突变、小片段插入或缺失为主，也可有整个外显子或基因的缺失^[10, 11]^。*LKB1*基因的突变使LKB1蛋白丧失了激酶活性，从而失去对细胞生长的控制，导致肿瘤的发生^[12]^。研究显示，绝大多数散发性肿瘤中，*LKB1*体细胞突变是罕见的^[13]^，但在非小细胞肺癌(non-small cell lung cancer, NSCLC)中，*LKB1* 的突变率可高达15%-35%^[14]^。LKB1可参与细胞内多种生物活动，在控制和调节细胞能量代谢、细胞增殖、细胞周期、细胞凋亡和细胞极性中发挥着重要作用^[15]^。将外源性*LKB1*基因导入无LKB表达的HeLa(宫颈癌细胞)和G361(恶性黑色素瘤细胞)中，可导致p21蛋白表达的增加，使细胞周期阻滞于G_1_期，抑制了细胞的增生^[16]^。Ji等^[17]^发现，LKB1在肺癌的发生起始、分化、转移中起着至关重要的作用。关于LKB1信号传导途径，目前了解的并不多。Alessi等^[18]^报道，LKB1可以磷酸化AMPKa亚单位活化环上172位点的苏氨酸，从而激活AMPK。

## 2 LKB1是AMPK的上游激酶

AMPK是一个异源三聚体蛋白，由一个具有催化活性的α亚单位及两个具有调节功能的β和γ亚单位组成，各个亚单位又可进一步分为α1、α2和β1、β2，γ1、γ2、γ3，不同的亚单位自由排列组合可以形成多种不同的AMPK三聚体^[19]^。α亚单位具有催化活性，有2个功能区，N端含有催化区域，是活性的核心部位，C末端含有与β和γ亚单位结合的区域，负责活性的调节；β亚基有2个相同的保守区域，分别为KIS和ASC区域。研究^[20]^表明，哺乳动物的ASC区域参与α和γ亚基的结合，KIS区域可能是一个糖原结合区，主要参与糖原的结合，对异源三聚体的定位非常重要；γ亚基含有4个串行重复的区域，命名为CBS(cystathionine beta synthase)区域，CBS在C端有2个这样的重复区域，每个区域约有60个氨基酸残基，它们借疏水作用力结合在一起，是AMP的结合位点。

AMPK是哺乳动物细胞中高度保守的蛋白质，是细胞的“代谢和能量感受器”。它对细胞内AMP/ATP比值变化相当敏感，在各种应激(缺氧、缺血、营养物质缺乏、运动等)下，AMP/ATP比值增加，AMPK活化，通过下调合成代谢过程(如蛋白质、脂肪酸和胆固醇的合成)减低ATP的消耗，同时促进催化氧化过程(如脂肪酸氧化、糖酵解等)以生成更多的ATP，缓解应激，维持机体的正常代谢^[21]^。

研究^[22, 23]^指出，应用AMPK激动剂，AICAR(5-氨基-4咪唑甲酰胺核苷酸)和苯乙双胍，处理HT1080(纤维肉瘤细胞）和*LKB1*^+/+^MEF（小鼠胚胎成纤维细胞）细胞，可以使AMPKa亚单位活化环上172位点的苏氨酸磷酸化（p-AMPK-a-Thr^172^），AMPK活化，而HeLa（不表达LKB1）和*LKB1*^-/-^MEF则无AMPK的活化；如果将野生型的*LKB1*基因导入HeLa和*LKB1*^-/-^MEF细胞后，再给于AICAR等处理，可以观察到AMPK的活化。Zhong等^[24]^应用AMPK激动剂，2-脱氧葡萄糖（2-deoxyglucose, 2-DG），处理*LKB1*野生型NSCLC细胞系H1299和H1792后，观察到p-AMPK-a-Thr^172^升高，而在*LKB1*基因突变细胞系A549、H460中，p-AMPK-a-Thr^172^无改变。上述研究提示LKB1是AMPK的上游激酶。此外，Karuman等^[3]^报道，在HT1080、IEC16(上皮细胞)和MEF等细胞中，LKB1可诱导细胞凋亡，并与P53蛋白功能有关。但张霞等^[25]^研究显示，*P53*突变或缺失并未影响到LKB1对AMPK的磷酸化作用，即LKB1-AMPK信号传导途径的调节与*P53*基因无关，所以LKB1与P53之间的信号传导联系还不是非常明确，有待进一步研究。

AMPK除了在调节能量代谢方面起重要作用外，活化的AMPK还可以磷酸化结节性硬化复合物(tuberous sclerosis complex)TSC1-TSC2，TSC复合物可抑制小GTP酶Rheb(Rashomolog enriched in brain)，而后者是mTOR活化所必需的刺激蛋白。在缺氧、营养匮乏等应激下，LKB1通过激活AMPK，进而磷酸化TSC1-TSC2，抑制Rheb的活性，负向调控mTOR的功能^[26]^。

## 3 LKB1-AMPK负向调控mTOR的功能

mTOR蛋白是一种非典型的丝氨酸/苏氨酸蛋白激酶，是磷脂酰肌醇32激酶相关激酶(phosphatidylinositol 32 kinase related kinase, PIKK)蛋白家族之一。mTOR蛋白的FRB(FKBP12-rapamycin binding)激酶结构域是雷帕霉素(rapamycin)的结合位点，当雷帕霉素与FRB区域结合后，可以抑制mTOR的激酶活性。

mTOR对生长因子、胰岛素、营养物质、氨基酸、葡萄糖等刺激产生应答，在调节细胞生长、增殖、调控细胞周期等多个方面扮演着重要角色。细胞受到生长因子等刺激后，磷脂酰肌醇3-羟基激酶(PI3K)活化，磷酸化其底物3, 4二磷酸磷脂酰肌醇(PIP2)转化成3, 4, 5三磷酸磷脂酰肌醇(PIP3)，进而通过磷脂酰肌醇依赖性激酶1、2(PDK1、PDK2)磷酸化Akt(蛋白激酶B)第308位点上的苏氨酸(Thr^308^)和第473位点上的丝氨酸(Ser^473^)，正向调节mTOR的功能^[27]^。活化的mTOR主要通过磷酸化蛋白翻译过程中的核糖体蛋白S6激酶(ribosomal protein S6 kinases, S6K)和真核细胞翻译启始因子4E结合蛋白1(the eIF4E-binding protein1, 4E-BP1)来控制细胞生长，二者是蛋白翻译的关键调节因子。S6K第389位苏氨酸可直接被mTOR磷酸化，磷酸化的S6K (p-S6K)可以促进延长因子-1a(elongation fator-1a, EF1a)、poly(A)结合蛋白等蛋白质翻译及表达^[28]^。S6K在多种人类肿瘤中呈高表达，S6K高表达的肿瘤预后较差^[29]^。eIF4E(eukaryotic translation initiation factor 4E)是真核细胞翻译启始复合体的亚单位之一，可与eIF4G结合，二者的结合受到4E-BPs的调节。低磷酸化的4E-BP1与eIF4E具有较高的亲和力，而处于高磷酸化状态的4E-BP1则可释放出eIF4E，使其与eIF4G结合，进而启动的5' cap mRNA的翻译。所以，当4E-BP1经mTOR作用发生磷酸化后，磷酸化的4E-BP1与elF4E发生分离，解除了翻译起始的抑制作用，从而增加了细胞周期蛋白D1、Rb蛋白、低氧诱导因子-1(hypoxia inducible factor-1, HIF-1)、血管内皮生长因子(vascular endothelial growth factor, VEGF)、CLIP-170等一组促进细胞生长关键蛋白的翻译，利于细胞的生长。目前已知，人的4E-BP1磷酸化位点有7个，分别是Thr37、Thr46、Ser65、Thr70、Ser83、Ser101和Ser112^[30]^。在生长因子的刺激下，mTOR首先磷酸化4E-BP1的Thr37和Thr46，进而磷酸化Thr70和Ser65。其中Thr70和Ser65的磷酸化对于eIF4E的释放最为重要，Thr70促进释放，而Ser65可以防止二者重新结合^[31]^，Zhou等^[32]^研究发现，mTOR、4E-BP1的磷酸化水平从正常乳腺上皮组织、不典型增生到恶性转化再到肿瘤浸润渐次增加，4E-BP1磷酸化水平越高，预后越差。所以mTOR信号通路的过度活化与肿瘤的发生、发展密切相关。

研究^[2]^表明，在能量短缺时，LKB1通过激活AMPKTSC2，抑制mTOR，抑制S6K和4E-BP1的磷酸化。在*LKB1*^-/-^MEF中，*LKB1*基因功能丧失，mTOR信号高度活化，S6K和4E-BP1磷酸化水平增高；而在HeLa(无LKB1表达)细胞中重新导入野生型*LKB1*，恢复LKB1的功能，可以观察到S6K和4E-BP1磷酸化水平的下降。另有研究^[33]^证明，应用AMPK激动剂(AICAR)注射大鼠腓肠肌可导致mTOR Ser^2448^、S6K Thr^389^、4E-BP1 Thr^37^位点磷酸化水平显著下降，其结果抑制了mTOR活性和蛋白质合成，阻止新生小鼠心肌肥厚。Zhong等^[24]^应用AMPK抑制剂(Compound C)预处理NSCLC细胞系H1299，可以阻止AMPK激动剂(2-DG)引起的S6KThr^389^位点磷酸化水平减低。综上所述，LKB1通过AMPK实现对mTOR的负向调控。

## 4 结语和前景

LKB1-AMPK-mTOR信号通路在调节细胞代谢、生长、增殖和凋亡中发挥着重要作用，*LKB1*的突变失活可导致mTOR信号通路异常活化，从而促进肿瘤的发生和发展。由于*LKB1*突变率在NSCLC中高达15%-35%，因此在肺癌中对该信号通路进行深入的探索是有意义的，可能为肺癌的靶向治疗提供新的思路。
